# Sensing the danger in mosquito spit

**DOI:** 10.1038/s44318-024-00073-w

**Published:** 2024-03-18

**Authors:** Yonca Keskek Turk, Liam D Barningham, Clive S McKimmie

**Affiliations:** Virus Host Interaction Team, School of Medicine, University of Leeds, Leeds, UK

**Keywords:** Immunology, Microbiology, Virology & Host Pathogen Interaction

## Abstract

Recent study identifies AaNRP as an arboviral infection-promoting factor in *Aedes aegypti* mosquito saliva that promotes recruitment of virus-susceptible myeloid cells.

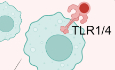

## Teaser

Factors contained within mosquito saliva enhance transmission of mosquito-borne viruses to the mammalian host. In this issue, Wang et al (2024) identify AaNRP as a crucial pro-viral factor in *Aedes aegypti* mosquito saliva that promotes recruitment of Zika and dengue virus-susceptible myeloid cells.

Mosquito-borne viruses represent an increasing threat to human health. The incidence of infection with these viruses, such as the Zika virus (ZIKV), dengue virus (DENV), and chikungunya virus (CHIKV), have significantly increased, infecting many millions each year. Although infection by these viruses is often asymptomatic or mild, many infections can result in severe disease or death. ZIKV is particularly noteworthy in its ability to cause severe defects in the developing nervous system of gestating embryos in pregnant mothers. The ongoing climate crisis combined with increased globalization of trade and transport is conspiring to put more people at risk of infection, and yet we have few vaccines, and no specific antiviral medicines are licensed for their treatment (Weaver et al, 2018). There are several challenges faced by those developing virus specific anti-virals. This includes the tendency of these viruses to emerge and re-emerge unpredictably and that each outbreak may be driven by a genetically distinct virus and/or serotype. Consequently, the development of a pan-viral medicine effective against multiple mosquito-borne virus species infections would be highly advantageous.

Anyone who has been bitten by a mosquito knows that this results in an itchy, inflamed swelling. It is into this unique inflammatory niche that mosquitoes transmit virus as they search for a blood meal. This is important for defining the severity of infection, as the host response to mosquito bites, and the saliva deposited, significantly enhances infection with these viruses (Pingen et al, 2016, 2017). This is a crucial stage of infection, as bitten skin is where the virus must replicate to enable efficient dissemination to the blood and remote tissues. An exciting concept that has gained ground in recent years is the therapeutic targeting of mosquito saliva components, or the bite itself, as a putative pan-viral therapy (Marin-Lopez et al, 2021; Bryden et al, 2020).

**Figure 1:**
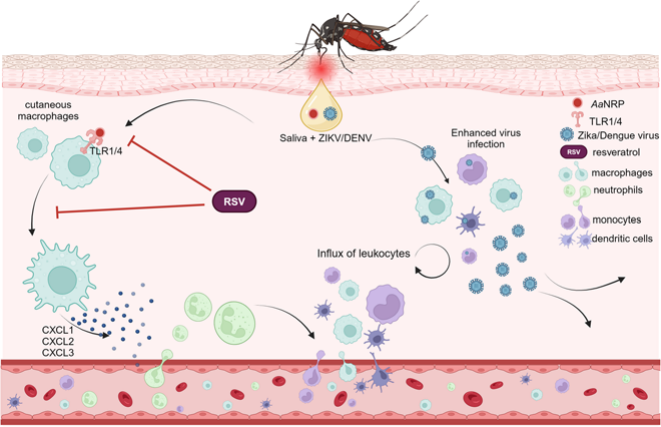
A simplified cartoon depicting the findings from Wang et al. Mosquito saliva contains multiple proteins including AaNRP, which is deposited into the dermis during biting. AaNRP activates TLR1 and TLR4-MyD88-NF-KB signaling in skin resident macrophages, resulting in the expression of leukocyte-recruiting chemokines. If virus, such as ZIKV, is present, some of these infiltrating myeloid cells become infected, release new infectious virus and make outcome of infection substantially worse. Selective inhibition of the MyD88 pathway by resveratrol inhibited AaNRP function. Created with Biorender.com.

Mosquito saliva contains a complex mixture of factors, some of which have evolved to enable highly efficient blood feeding. Intriguingly, a number of these factors also enhance the infection of the vertebrate host with virus. These factors include sialokinin (Lefteri et al, 2022; Martin-Martin et al, 2022) which acts within seconds to permeabilise blood vessels and promote fluid and leukocyte entry; NeSt1 which activates neutrophils (Hastings et al, 2019); AaVA1 which activates autophagy (Sun et al, 2020); and LTRIN that suppresses lymphotoxin-beta function (Jin et al, 2018). These findings have helped define which factors in mosquito saliva are responsible for inducing the rapid and robust influx of leukocytes that occurs following mosquito biting. Some of these cells are susceptible to infection and replicate virus to higher quantities than would otherwise occur if virus was deposited in uninflamed, non-bitten skin (Pingen et al, 2017). However, most mosquito salivary factors remain poorly characterized, and their role in modulating host susceptibility to virus infection not studied. In a recent publication, Wang et al (2024) report a partial screen of mosquito salivary factors leading to the identification of the novel, pro-inflammatory salivary factor AaNRP. Fascinatingly, AaNRP directly activates innate immune sensors on skin resident macrophages, thus activating an inflammatory program that recruits leukocytes into the skin. These newly recruited cells provide new cellular fodder for viral replication and thereby enhance infection (as summarized in Fig. 1).

To begin with, Wang et al use a non-biased screening-based approach to assess the ability of 25 different salivary proteins to recruit neutrophils into the skin. Neutrophils are rapidly recruited during mosquito biting and serve to coordinate localized inflammatory response that recruits virus-permissive cells such as dendritic cells, monocytes, and macrophages. Injection with three of these salivary proteins, particularly AaNRP, induced similar responses in the skin to a mosquito bite and resulted in leukocyte recruitment. Similarly, depletion of AaNRP expression in mosquito salivary glands limited the ability of mosquito biting to induce inflammatory responses in skin. The entry of neutrophils appeared crucial, as in their absence AaNRP did not induce inflammation.

Next, they assessed how AaNRP could act to recruit neutrophils. Firstly, they discounted the ability of AaNRP to be chemotactic through use of transwell assays. Surprising, they also found mast cells, which are required for many immune responses to allergens and other immunogens, were not significantly activated by mosquito biting. Instead, skin bitten by AaNRP-sufficient mosquitoes, or injected with purified AaNRP, activated skin resident macrophages directly to initiate a chemokine centric program that recruited neutrophils and other innate immune leukocytes. Specifically, they used single-cell sequencing to define high expression of neutrophil-attracting chemokines CXCL1-3 in macrophages. Depletion of macrophages limited the ability of skin to recruit neutrophils following biting, suggesting an essential role in this process. To define the molecular mechanism by which AaNRP activated macrophages, they assessed whether the well-described canonical activator of chemokine expression, MyD88/NF-κB signaling, was necessary. Interestingly, luciferase-based assays showed strong induction of the canonical MyD88-dependent pathway by AaNRP, an effect that was reversed by a MyD88-specific inhibitor. To define which receptor could be involved in activating MyD88, they performed immunoprecipitation assays to show direct binding of AaNRP specifically to TLR1 and TLR4, but not other TLRs. TLR1/4 inhibitors also prevented AaNRP-mediated expression of chemokine, while a knockdown of TLR1 and TLR4 aborted NF-κB signaling by AaNRP. Together this demonstrates for the first time that a mosquito-derived salivary factor is a potent activator of mammalian TLR signaling in skin macrophages, and that this initiates a chemokine-associated inflammatory response that recruits leukocytes.

To define whether AaNRP activation of TLRs modulated host susceptibility to virus infection, they next inoculated mice with ZIKV in the presence or absence of AaNRP. Following ZIKV infection, AaNRP robustly recruited virus-permissive myeloid cells, some of which some became infected and released new infectious virus, resulting in more efficient virus dissemination and worse infection in a MyD88 pathway-dependent manner. Similar results were found following infection with the dengue virus. Impressively, they also showed that ZIKV-infected mosquitoes, when allowed to bite mice, were less able to induce high titer viremia and disease in mice, if depleted for AaNRP.

Although AaNRP is unlikely to have evolved for the specific purpose of activating TLR signaling in mammalian skin, these data nonetheless show it is unambiguously inflammatory and that this host response significantly enhances infection by the virus. This is particularly intriguing as mosquitoes that lacked AaNRP had normal skin probing times and the ability to engorge with blood. As such, it does pose the question why mosquitoes would have evolved to express AaNRP at all, as the ability to enhance virus infection in vertebrates would not have asserted any evolutionary pressure in this respect.

In the final chapter of this interesting story, the authors ask whether therapeutic targeting of AaNRP-induced signaling can limiting the efficient establishment of virus in the skin following bite by an infected mosquito. Such an approach is not unprecedented, as mice vaccinated against saliva factors NeSt1 and AgBR1 (Marin-Lopez et al, 2021) exhibit some protection from mosquito-transmitted ZIKV infection. Here, Wang et al show that it is possible to limit AaNRP-mediated enhancement of ZIKV infection through use of the well-described MyD88-NF-κB pathway inhibitor resveratrol. This interesting compound is found naturally, e.g., in red grapes. Here, administration of resveratrol to mice limited chemokine expression and leukocyte recruitment to AaNRP injection and mosquito biting. In doing so, this afforded a substantial degree of protection from ZIKV infection, as observed through decreased virus quantities, ability to disseminate and cause disease.

Together these data help solidify the concept that host responses to mosquito saliva that promotes rapid and robust leukocyte entry to bitten skin are central for its pro-viral capacity. A key step in this mechanism has now been defined with the identification of AaNRP as a crucial pro-viral factor in *Aedes aegypti* mosquito saliva. This species of mosquito is responsible for transmitting most arbovirus infections worldwide and constitutes a new target that coud limit the development of disease caused by a wide number of these genetically diverse viruses. This proof-of-concept study uses resveratrol to limit AaNRP function, which, if extrapolated for limiting the development of disease in humans, would most likely require prophylactic and routine dosing. As such, careful consideration, and further pre-clinical testing, would be needed to define the potential efficiency and side effects of such putative AaNRP inhibitors before devising trials in humans. Although seemingly controversial, such a prophylactic approach is common in other contexts, such as the use of aspirin for those at high risk of heart disease. Similarly, outbreaks of mosquito-borne disease are highly seasonal. Those individuals, such as the immunosuppressed that are at higher risk during these periods, may benefit from such a strategy; only time and further studies will tell if such an approach is effective and safe.
